# Periapical bone edema volume in 3D MRI is positively correlated with bone architecture changes

**DOI:** 10.1186/s13244-025-01903-z

**Published:** 2025-01-29

**Authors:** Alexander W. Marka, Monika Probst, Tobias Greve, Nicolas Lenhart, Niklas Graf, Florian Probst, Gustav Andreisek, Thomas Frauenfelder, Matthias Folwaczny, Egon Burian

**Affiliations:** 1 Department of Diagnostic and Interventional Radiology, Klinikum rechts der Isar, School of Medicine, Technical University of Munich, Munich, Germany; 2 Department of Diagnostic and Interventional Neuroradiology, Klinikum rechts der Isar, School of Medicine, Technical University of Munich, Munich, Germany; 3 Department of Neurosurgery, LMU University Hospital, LMU Munich, Munich, Germany; 4 Department of Oral and Maxillofacial Surgery, Helios Clinic Munich West, Teaching Hospital of the Ludwig-Maximilians-University of Munich, Munich, Germany; 5 Department of Oral and Maxillofacial Surgery, University of Munich, Munich, Germany; 6 Department of Diagnostic and Interventional Radiology, Cantonal Hospital Frauenfeld, Frauenfeld, Switzerland; 7 Diagnostic and Interventional Radiology, University Hospital of Zurich, University Zurich, Zurich, Switzerland; 8 Department of Restorative Dentistry and Periodontology, LMU University Hospital, Ludwig-Maximilians-University, Munich, Germany

**Keywords:** Magnetic resonance imaging, Periapical osteolysis, Dentistry, Dental radiographs

## Abstract

**Objectives:**

To compare and correlate bone edema volume detected by 3D-short-tau-inversion-recovery (STIR) sequence to osseous decay detected by a T1-based sequence and conventional panoramic radiography (OPT).

**Materials and methods:**

Patients with clinical evidence of apical periodontitis were included retrospectively and received OPT as well as MRI of the viscerocranium including a 3D-STIR and a 3D-T1 gradient echo sequence. Bone edema was visualized using the 3D-STIR sequence and periapical hard tissue changes were evaluated using the 3D-T1 sequence. Lesions were segmented and volumes were calculated for bone edema and structural decay. OPTs were assessed for corresponding periapical radiolucencies using the periapical index (PAI).

**Results:**

Of the 42 patients of the initial cohort 21 patients with 38 periapical lesions were included in the analysis (mean age 57.2 ± 13.8 years, 9 women). Reactive bone edema was detected on MRI in 23 periapical lesions with corresponding radiolucency on OPT. Fifteen periapical lesions were detected only in the STIR sequence. The volume of edema measured in the STIR was significantly larger in OPT-positive lesions (mean: STIR (OPT+) 207.3 ± 191.1 mm³) compared to OPT-negative lesions (mean: STIR (OPT−) 29.5 ± 34.2 mm³, *p* < 0.001). The ROC curve analysis demonstrated that Volume T1 (0.905, *p* < 0.01) and Volume STIR (0.857, *p* < 0.01) measurements have strong diagnostic performance for distinguishing OPT-positive from OPT-negative lesions.

**Conclusion:**

Clinically symptom-free patients without pathologic changes in OPT can show signs of inflammation within the periapical bone. Bone edema volume visualized by STIR sequence exceeds bone architecture changes indicated in T1-based imaging and might precede osteolysis in dental radiography.

**Critical relevance statement:**

These results show that subtle intraosseous inflammation within the periapical tissue might remain undetected by conventional dental radiography and T1-based sequences. This emphasizes the potential of MRI in secondary prevention in dentistry.

**Key Points:**

- Conventional panoramic radiography (OPT) may show only delayed findings of pathological periapical changes.
- MRI detected bone edema in 23 radiolucent lesions on OPT.
- MRI revealed 15 lesions only visible with STIR sequences.
- STIR sequences showed bone inflammation undetectable by conventional radiography or T1 imaging.
- MRI offers diagnostic advantages for early dental pathology detection.

**Graphical Abstract:**

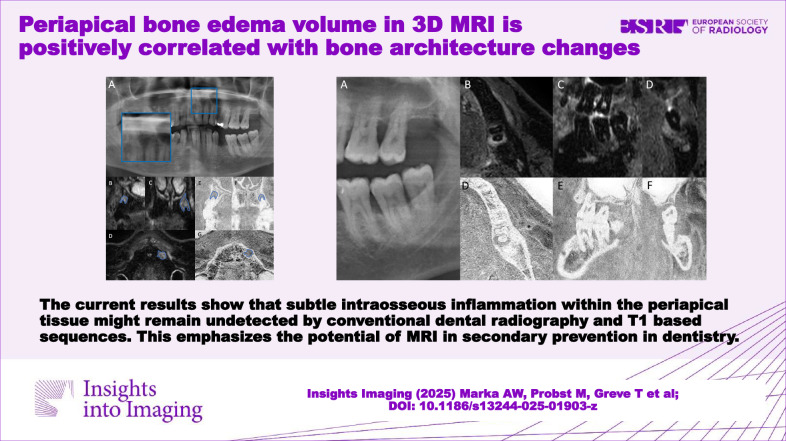

## Introduction

Until now the diagnosis of apical periodontitis (AP) is solely based on clinical findings and dental radiography. Conventional panoramic radiography (OPT) is one of the basic dental imaging modalities available in every dental practice and an integral component of the routine diagnostic workup in daily routine. However, recent studies have shown that pathologic periapical changes might be visible in conventional imaging with considerable delay compared to cross-sectional imaging like cone beam computed tomography (CBCT) and magnetic resonance imaging (MRI) [1, 2]. The underlying pathophysiological mechanisms are complex and range from non-specific inflammatory reaction in the periapical region to periapical bone resorption and distinct radiolucent lesions seen on dental radiography [3–5]. In terms of secondary prevention in conversative dentistry, early detection of AP is crucial for timely initiation of corresponding treatment and avoiding complications, as seen in osteomyelitis or sinusitis [5–7].

Recently, MRI has been implemented not only in multiple dental disciplines like oral surgery, periodontology and orthodontics [2, 8–12]. The benefit of MRI was confirmed also for the detection of clinically inapparent inflammation in odontogenic maxillary sinusitis [5]. Furthermore, the use of MRI in visualizing subtle inflammation processes in tooth-supporting alveolar bone and the gingival soft tissue receives increasing attention [11, 13–17]. While MRI has proven beneficial in various dental disciplines, ultrasound, another radiation-free imaging modality, has also demonstrated significant potential, particularly in the differentiation of periapical lesions, as shown in recent studies [18].

Two recent narrative reviews published by Flügge et al and Greiser et al highlighted the great potential of MRI in dentistry and emphasized its benefit when used complementary to conventional radiation-based diagnostic procedures and cross-section modalities [19, 20]. Still, the mainstay of clinical dental imaging is built on radiography and CBCT and the use of MRI is still reserved for more complex cases coming along with extensive soft tissue involvement [21].

Increasing evidence on the capabilities of MRI regarding early detection of inflammation within the gingiva, the periodontal ligament, the alveolar bone and even dental hard tissue is available [1, 2, 16, 22, 23]. However, only insufficient quantitative data on the volume and the composition of the bone edema associated with periapical osteolysis have been reported so far, giving detailed insight into the pathophysiology of periapical inflammation.

The aim of this study was to determine the volume of bone edema and analyze structural changes of periapical lesions in MRI and correlate the observations with findings in OPTs. Based on these data cutoff values specific for structural changes in MRI should be identified. Finally, the extent of structural changes within the periapical and the alveolar bone remaining undetected with conventional diagnostic imaging should be delineated.

## Materials and methods

### Patient selection

Patients with clinical signs of periodontal disease attending the Department of Restorative Dentistry and Periodontology, LMU University Hospital, Ludwig-Maximilians-University Munich, for periodontal treatment, were prospectively enrolled between March 2018 and April 2019. For this retrospective analysis, 38 teeth from 21 patients (mean age 57.2 ± 13.8 years, 9 women) were considered. All clinical assessments were conducted by experienced dentists. For this study, only teeth with periapical changes on MRI were included which showed no clinical signs of pulpitis. The MRI examiners were blinded to clinical findings and vice versa. The study adhered to the STROBE guidelines for observational research and was conducted in compliance with the principles of the Declaration of Helsinki. Written informed consent was obtained from all participants. The study protocol received approval from the institutional review boards of the Technical University of Munich (Ref.-No. 185/18 S) and Ludwig-Maximilians-University Munich (Ref.-No. 18-657). The study was also retrospectively registered with the German Clinical Trials Register (DRKS00020761).

### MRI acquisition

All participants underwent MRI scans using a 3-Tesla scanner (Ingenia; Philips Healthcare, Best, The Netherlands) equipped with a specialized 16-channel head-neck and spine coil (dStream Head Neck Spine coil, Philips Healthcare). The imaging protocol included a 3D STIR sequence and a 3D isotropic fast field echo (FFE) T1-weighted sequence (see Table 1). Images were obtained in the axial plane and subsequently reformatted into sagittal and coronal orientations.

**Table 1:** MRI sequences

Variables	3D STIR	3D isotropic fast field echo T1
TR (ms)	2300	10
TE (ms)	184	1.75
Acq. resolution (mm)	0.65 × 0.65	0.43 × 0.43
Slice thickness (mm)	1	0.43
Number of slices	180	180
Acceleration factor	2.5	xx
Scan time (min.)	6:03	5:32

*TR* repetition time, *TE* echo time, *STIR* short tau inversion recovery

### OPT acquisition

Orthopantomograms (OPTs) were acquired using a 2D X-ray device (Orthopos S 2D; Dentsply Sirona, Charlotte, NC, USA) with an exposure time of 14.1 s, set to 63 kV and 8 mA.

### Image analysis

The 3D STIR and T1 FFE sequences were evaluated for bone changes associated with periapical lesions. Bone edema was assessed using the STIR sequence indicating inflammatory changes. Bone architecture deterioration was evaluated using the T1 FFE sequence. Each sequence was reconstructed in transverse, sagittal, and coronal planes. Detected lesions were segmented, and the volume of signal alterations was quantified. Periapical radiolucencies on OPTs were assessed using a modified periapical index (PAI) score, ranging from 1 (healthy) to 5 (severe periapical osteolysis), based on criteria from Gürhan et al [24]. Definitions of the PAI score are shown in Table 2. The signal-to-noise ratio (SNR) was analyzed according to the formula below. Detailed mathematical expressions are presented in the Supplementary material S1.

**Table 2:** Periapical index (PAI) for panoramic and apical radiographs

PAI	Definitions
1	Normal periapical structures
2	Small changes in bone structure
3	Changes in bone structure with mineral loss
4	Apical periodontitis with well-defined radiolucent area
5	Severe apical periodontitis with exacerbating features

SNR Formula:

$$\left[{{\rm{SNR}}}=\frac{{\mu }_{{{\rm{Lesion}}}}-{\mu }_{{{\rm{Muscle}}}}}{{\sigma }_{{{\rm{Lesion}}}}}\right]$$

Where:

- ${\mu }_{{{\rm{Lesion}}}}$ = Mean Signal Intensity of the Lesion
- ${\mu }_{{{\rm{Muscle}}}}$= Mean Signal Intensity of the Muscle (Masseter muscle)
- ${\sigma }_{{{\rm{Lesion}}}}$ = Standard Deviation of the Signal Intensity of the Lesion

All image analyses, including MRI, dental radiographs, and OPTs, were conducted by a radiology resident (A.W.M.) and a dentist (N.G.), both with 3 years of experience. Images were analyzed independently and blinded to other diagnostic information. In cases of significant artifacts, such as those from metallic restorations or motion, affected teeth were excluded from analysis. Analyses and segmentations were performed using a certified picture archiving and communication system workstation (IDS7 21.2; Sectra, Linköping, Sweden). MRI and OPT images were reviewed at least 8 weeks apart to ensure independent evaluations. For intra-reader agreement, ten patients were reassessed after 8 weeks by both raters.

### Statistics

Statistical analyses were performed using SPSS Version 29.0.0.0 (IBM Corp., Armonk, NY). Pearson correlation coefficients were computed to assess the linear relationships between variables, as this method is appropriate for measuring the strength and direction of associations between two continuous variables. Intraclass Correlation Coefficient (ICC) was used to compare inter-reader variability, providing a measure of the consistency or reproducibility of quantitative measurements made by different observers.

Group differences were evaluated using the Mann–Whitney U test due to the non-parametric nature of the data, which did not assume a normal distribution and was suitable for comparing differences between two independent groups. Receiver Operating Characteristic (ROC) curves were generated to evaluate the diagnostic performance of the predictive models. The Youden Index was calculated to determine the optimal cutoff points on the ROC curves, maximizing the sum of sensitivity and specificity. Statistical significance was set at *p* < 0.05 for all analyses.

## Results

### Patient demographics

A total of 42 patients were initially enrolled in the study. Among them, 21 patients with 38 periapical lesions were included in the final analysis. The mean age of the patients was 57.2 ± 13.8 years, and the cohort included 9 women.

### Detection and volume comparison

The mean volumes measured in STIR and T1 sequences are presented in Table 3. Among the 38 periapical lesions assessed, 23 lesions showed reactive bone edema on STIR sequences and bone alterations in T1 sequences with corresponding radiolucency on OPT. In contrast, 15 lesions were detected by the STIR sequence only but not the OPT. The volume of edema in the STIR sequence was significantly larger in OPT-positive lesions (mean: 207.3 ± 191.1 mm³) compared to OPT-negative lesions (mean: 29.5 ± 34.2 mm³, *p* < 0.001). Similarly, the lesion volume measured in T1 was significantly larger in OPT-positive lesions (mean: 109.3 ± 124.3 mm³) compared to OPT-negative lesions (mean: 1.9 ± 5.1 mm³, *p* < 0.001). This finding is consistent with the observation that lesions absent in T1 are also absent in OPT. The SNR was analyzed, showing a significant difference between OPT-positive and OPT-negative lesions for SNR-STIR (*p* = 0.002), but not for SNR-T1 (*p* = 0.332) (see Table 3).

**Table 3:** Results of volumetry and SNR

Group	Volume STIR (mm³)	Volume T1 (mm³)	SNR-STIR	SNR-T1
All lesions (*N* = 38)	138.0 ± 172.6	66.9 ± 109.6	1.34 ± 0.53	0.70 ± 1.35
OPT-positive lesions (*N* = 23)	207.3 ± 191.1	109.3 ± 124.3	1.59 ± 0.11	0.78 ± 0.30
OPT-negative lesions (*N* = 15)	29.5 ± 34.2	1.9 ± 5.1	1.31 ± 0.04	−0.18 ± 0.70
*p*-value	< 0.001	< 0.001	0.002	0.332

Data are presented as means ± standard deviation

## Correlations

**Fig. 1:**
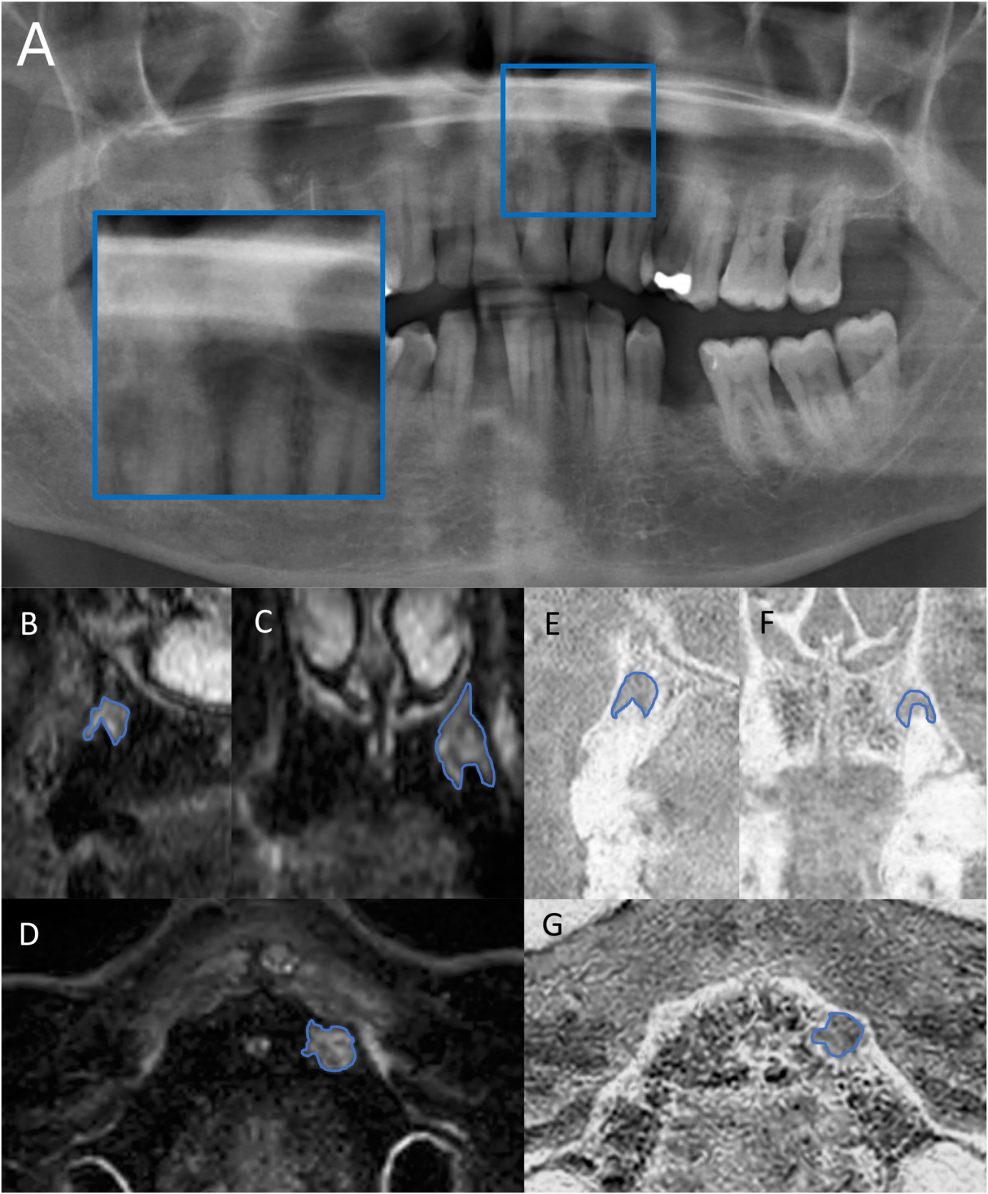
3D-segmentation of a periapical bone marrow edema of tooth 22 in a 64-year-old man. A periapical radiolucency is present in OPT (**A**) and was rated as PAI 5. Sagittal (**B**), coronal (**C**) and axial (**D**) reconstructions of a 3D STIR sequence of the same tooth showing a bright periapical edema at tooth 22 with a volume of 200 mm^3^. Sagittal (**E)**, coronal (**F)** and axial (**G)** reconstructions of a 3D T1 sequence showing a periapical bone defect with a volume of 146 mm^3^ at the root of tooth 22

**Fig. 2:**
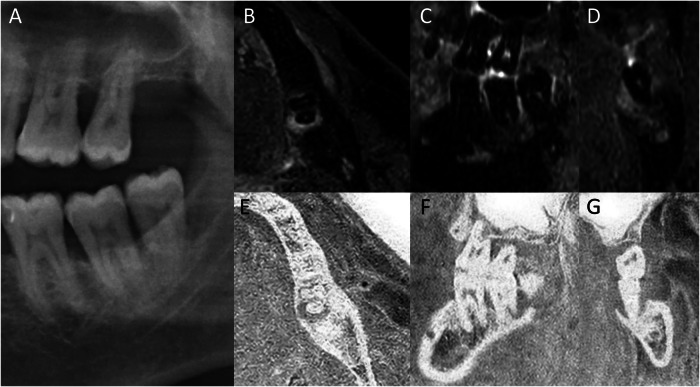
63-year-old male patient with a periapical lesion of the distal root of tooth 37. No periapical radiolucency (thick white arrow) is visible in OPT (**A**). The axial (**B**), sagittal (**C**) and coronal (**D**) reconstructions of a 3D STIR sequence show a large bone marrow edema around a periapical lesion, with a volume of 127 mm^3^ and a signal-to-noise ratio of 2,16. The axial (**E**), sagittal (**F**) and coronal (**G**) reconstructions of a 3D T1 sequence show a corresponding small bone defect with a volume of 65 mm^3^

The Pearson correlation coefficient between volume of bone marrow edema measured in STIR and volume of intraosseous lesions measured in the T1 sequence was 0.794 (*p* < 0.001), indicating a strong positive correlation (see Table 4). However, there was no significant correlation between Volume T1 and SNR-T1 (*r* = 0.113, *p* = 0.608) or between Volume STIR and SNR-STIR (*r* = 0.107, *p* = 0.524). Significant correlations were found between PAI ratings and volume measurements. PAI rating was significantly correlated with Volume STIR (*r* = 0.583, *p* < 0.001), Volume T1 (*r* = 0.630, *p* < 0.001), and SNR-STIR (*r* = 0.328, *p* = 0.044), indicating a strong relationship between the severity of radiographic periapical inflammation and both MRI, the volume and slightly less the MRI signal intensity. Figures 1 and 2 showcase clinical cases in which periapical lesions could be detected using MRI.

**Table 4:** Correlation of volumetry, SNR and PAI rating

Variable	Volume STIR	Volume T1	SNR-STIR	SNR-T1	PAI
Volume STIR	1	0.794**	0.107	0.425	0.583**
Volume T1	0.794**	1	0.125	0.113	0.630**
SNR-STIR	0.107	0.125	1	0.012	0.382*
SNR-T1	0.425	0.113	0.012	1	−0.137

Pearson’s correlation coefficients with *p ≤ 0.05 and **p ≤ 0.001

### ROC curve and Youden Index analysis

The ROC curve analysis revealed significant diagnostic performance for volume and SNR measurements in distinguishing detectable periapical lesions in OPT. Notably, the analysis proved to be particularly useful for distinguishing between PAI 1 versus PAI 2-5 (indicating osteolysis) and between PAI 5 (most severe form of osteolysis) versus PAI 1-4 to 4 (see Table 5).

**Table 5:** ROC and Youden Index for volumetry and SNR in regard to PAI rating

Variable	Volume STIR	Volume T1	SNR-STIR	SNR-T1
AUC for PAI 1 versus rest	0.857**	0.905**	0.726*	0.738
Youden Index	42.6 mm³	16.1 mm³	1.35	0.57
AUC or PAI 5 versus rest	0.824**	0.873**	0.529	0.549
Youden Index	234.5 mm³	117.5 mm³	1.41	1.22

AUC values for ROC with *p ≤ 0.05 and **p ≤ 0.001

*AUC* area under the curve

For PAI 1 versus other ratings, the area under the curve (AUC) was highest for Volume T1 (0.905, *p* < 0.01) and Volume STIR (0.857, *p* < 0.01), indicating strong discriminatory power. The AUC for SNR-STIR was 0.726 (*p* < 0.05), while the AUC for SNR-T1 was 0.738, which was not statistically significant. The highly significant AUC values (Volume T1 and Volume STIR) indicate these measurements are particularly useful for diagnostic purposes.

The Youden Index analysis identified the optimal diagnostic cutoff points. For PAI 1 versus rest, the Youden Index was 42.6 mm³ for Volume STIR, 16.1 mm³ for Volume T1, 1.35 for SNR-STIR, and 0.57 for SNR-T1, optimizing the balance between sensitivity and specificity.

Similarly, for PAI 5 versus other ratings, Volume T1 and Volume STIR exhibited high AUC values of 0.873 (*p* < 0.01) and 0.824 (*p* < 0.01), respectively, demonstrating significant diagnostic capability. The AUC values for SNR-STIR and SNR-T1 were lower at 0.529 and 0.549, respectively, and neither was statistically significant. The Youden Index for PAI 5 versus other ratings was 234.5 mm³ for Volume STIR, 117.5 mm³ for Volume T1, 1.41 for SNR-STIR, and 1.22 for SNR-T1.

These findings indicate the effectiveness of Volume T1 and Volume STIR measurements in the diagnostic evaluation of PAI, with Volume T1 consistently showing the highest AUC values and optimal cutoff points as indicated by the Youden Index. The significant and highly significant measurements, particularly Volume T1 and Volume STIR, are more useful for diagnostic purposes compared to those that are not statistically significant.

### Intraclass correlation coefficient (ICC)

The ICC values for all measured variables were within acceptable ranges, indicating good reliability. Specifically, the ICC for Volume STIR, Volume T1, and PAI Rating were all above 0.90, reflecting excellent reliability. The ICC for SNR-STIR was 0.81 and for SNR-T1 was 0.72, both representing good reliability.

## Discussion

The results of this study highlight the potential of MRI in early, quantitative detection of alveolar bone inflammation before the manifestation of structural osseous lesions. With this quantitative analysis of the composition and volume of intraosseous, periapical bone edema, we show that inflammation-associated changes within the tooth-supporting alveolar bone can remain undetected with means of conventional dental radiography. These changes can be reliably visualized with the water-sensitive STIR sequence already when initial edematous changes are present. According to the current data, the volume of bone edema was significantly correlated with the severity of lesions within the osseous matrix at the periapical region detected by T1-based imaging and OPT. Both findings are important for the preventive use of MRI in dentistry but also for stratifying disease extent and danger of exacerbation to adjacent structures like the sinus or the orbit in advanced cases [5, 25].

As indicated, routine dental clinical examinations are complemented with radiography. The first-line diagnostic modality is OPT. However, as shown by prior works of our group, there are changes with yet unknown significance in the periapical alveolar bone that cannot be reliably captured by dental radiography [1].

It has been commonly accepted for many years that the detection of intraosseous lesions using conventional dental radiography requires the involvement of either the lingual or the vestibular cortical structures or both [26]. Numerous qualitative and quantitative studies using cross-sectional imaging have correlated findings in CBCT with corresponding dental radiography. In these studies, the inherent limitations of dental radiography in the detection of subtle lesions or small anatomic structures were addressed in detail [27, 28]. As one potential consequence of missed lesions or anatomic structures, these cases remain at least partially untreated, particularly regarding accessory mesio-buccal root canals in maxillary molars or supernumerary canals in mandibular incisors leading to treatment failures [27]. Going a step further, not only the detection of subtle lesions itself is difficult in clinical routine but also the visualization of diffuse inflammation affecting the periapical bone and the adjacent soft tissue [5]. In this context, water-sensitive T2-based sequences can improve the visualization of pathogenic changes remaining undetected otherwise but being responsible for chronic pain [29–31]. Most of these cases are extremely frustrating for both patients and practitioners alike.

MRI seems to have the potential to enhance the diagnostic armamentarium of dentistry in the future. Hence, a profound understanding of the possibilities and limitations of different MRI-based dental imaging techniques might be increasingly interesting for clinicians. The understanding of the correlations of how pathologic findings might present in cross-sectional imaging while going undetected in conventional imaging will be essential for effective resource allocation and treatment success. Moreover, MRI could prove particularly valuable in cases of recurrent infections or infections of unknown origin, including severe cases involving the maxillary sinuses or meninges, where conventional imaging may fall short in revealing subtle underlying dental pathology.

The quantitative results of the present study further emphasize the preventive character of tooth-related MR images. Volumes of bone edema were significantly larger in OPT with radiolucencies compared to OPTs with negative findings. In previous studies, the diagnostic capabilities of MRI have been shown not only for the T1-based detection of subtle hard tissue changes in the bone but also in the enamel and dentin [32–34]. In prior works, we proved how water-sensitive sequences can complement the findings of hard tissue changes harbored in T1-based sequences in regard to caries detection, periodontitis and apical osteitis [1, 2, 23]. Herein, we further show how these findings correlate with each other.

The most promising aspect of our findings presents the implementation of dental MRI including the STIR sequence in early detection of otherwise hidden periapical inflammation. This is especially important in settings, where radiation or antiresorptive treatment is planned. Early detection and subsequent treatment might lower the risk of osteoradionecrosis of the jaw or medication-related osteonecrosis of the jaw. Further, the advantage of using water-weighed T2 imaging might not only lie in the prevention of disease exacerbation but also in the detection of the origin of severe inflammatory conditions like maxillary sinusitis or maxillary empyema to assure prompt and precise treatment initiation.

The current study has several limitations. First, the small sample size and the retrospective character of this study only allow for associations of different pathologic findings. Second, there were no periapical radiographs or CBCT available for the correlation of the MRI findings. While CBCT could provide more detailed information about osseous changes and bone resorption with higher sensitivity than apical radiographs and OPT, our study focused on comparing MRI with OPT as it is a more commonly used imaging technique in routine dental practice. Nonetheless, future studies incorporating CBCT could offer more comprehensive insights into bone changes, particularly osteolysis, and further validate MRI’s role in detecting early pathological changes in preventive dental imaging. Third, as there was no follow-up of the detected edematous periapical bone changes, there are no data on the further development of these changes. It is possible that the osseous changes detected in the STIR sequence are temporary and represent adaptive responses to biomechanical forces, such as stress reactions in the bone marrow. Persistent mechanical stress has been shown to cause hypointensity on T1 and corresponding STIR hyperintensity, indicating a stress response rather than a pathological lesion [35]. As such, the clinical significance of the detected edema without architectural changes in T1 and no osteolysis detected in OPT remains unclear. Further follow-up studies would be necessary to assess whether these changes resolve or progress over time.

Summarizing our results, we report that bone edema volume visualized by STIR sequence exceeds bone architecture changes as indicated in T1-based imaging and seems to precede osteolysis in dental radiography. A potential implementation of the STIR sequence in the dental imaging portfolio might improve early detection in preventive and conservative dentistry.

## Supplementary information

ELECTRONIC SUPPLEMENTARY MATERIAL

## Data Availability

Data are available upon request from the corresponding author.

## Abbreviations

AP: Apical periodontitis
CBCT: Cone beam computed tomography
FFE: Fast field echo
ICC: Intraclass correlation coefficient
MRI: Magnetic resonance imaging
OPT: Orthopantography
PAI: Periapical index
SNR: Signal-to-noise ratio
STIR: Short tau inversion recovery

## Declarations

### Study subjects or cohorts overlap

There is a partial overlap in the patient cohort with two previously published studies: 1. Schwarting et al [36]. 2. Probst et al [2]. In the *Insights into Imaging* paper, the research focused on detecting bone edema in apical periodontitis. However, our current study extends this by defining cutoff values for edema volume that can be detected in conventional imaging modalities, emphasizing the significance of water-sensitive MRI sequences for secondary prevention in dental practice. This novel approach provides crucial insights into the diagnostic thresholds that were not addressed in the previous studies.
