# Supplementary material for: Periapical bone edema volume in 3D MRI is positively correlated with bone architecture changes
Source: Insights Imaging. 2025 Jan 29;16:26. doi: 10.1186/s13244-025-01903-z (PMC11780240; doi:10.1186/s13244-025-01903-z)
Supplement: Supplementary file 1 — ELECTRONIC SUPPLEMENTARY MATERIAL [file 13244_2025_1903_MOESM1_ESM.pdf]

Periapical bone edema volume in 3D MRI is positively correlated with bone architecture changes

ELECTRONIC SUPPLEMENTARY MATERIAL

S1: Detailed Mathematical Expressions

1. Mean Signal Intensity of the Lesion (μ_{Lesion}):

$$\mu_{\text{Lesion}} = \frac{1}{N_{\text{Lesion}}} \sum_{i=1}^{N_{\text{Lesion}}} S_{\text{Lesion},i}$$

- N_{Lesion} = Number of pixels/voxels in the lesion region.

- $S_{\text{Lesion},i}$= Signal intensity of the i-th pixel/voxel in the lesion region.

2. Mean Signal Intensity of the Muscle (μ_{Muscle}):

$$\mu_{\text{Muscle}} = \frac{1}{N_{\text{Muscle}}} \sum_{j=1}^{N_{\text{Muscle}}} S_{\text{Muscle},j}$$

- N_{Muscle} = Number of pixels/voxels in the muscle ROI.

- $S_{\text{Muscle},j}$ = Signal intensity of the j-th pixel/voxel in the muscle ROI.

3. Standard Deviation of the Signal Intensity of the Lesion (σ_{Lesion}):

$$\sigma_{\text{Lesion}} = \sqrt{\frac{1}{N_{\text{Lesion}}} \sum_{i=1}^{N_{\text{Lesion}}} \left(S_{\text{Lesion},i} - \mu_{\text{Lesion}}\right)^2}$$

- This measures the variability of the signal intensities within the lesion region.
